# The acid sphingomyelinase/ceramide system in COVID-19

**DOI:** 10.1038/s41380-021-01309-5

**Published:** 2021-10-04

**Authors:** Johannes Kornhuber, Nicolas Hoertel, Erich Gulbins

**Affiliations:** 1 Department of Psychiatry and Psychotherapy, University Hospital, Friedrich-Alexander-University of Erlangen-Nuremberg, Erlangen, Germany; 2 AP-HP.Centre – Université de Paris, Département Médico-Universitaire de Psychiatrie et Addictologie, Hôpital Corentin-Celton, 92130 Issy-les-Moulineaux, France; 3 INSERM, Institut de Psychiatrie et Neurosciences de Paris, UMR_S1266, Paris, France; 4 Department of Molecular Biology, University of Duisburg-Essen, Essen, Germany; 5 Department of Surgery, University of Cincinnati, Cincinnati, OH USA

**Keywords:** Molecular biology, Diseases

## Abstract

Acid sphingomyelinase (ASM) cleaves sphingomyelin into the highly lipophilic ceramide, which forms large gel-like rafts/platforms in the plasma membrane. We showed that SARS-CoV-2 uses these platforms for cell entry. Lowering the amount of ceramide or ceramide blockade due to inhibitors of ASM, genetic downregulation of ASM, anti-ceramide antibodies or degradation by neutral ceramidase protected against infection with SARS-CoV-2. The addition of ceramide restored infection with SARS-CoV-2. Many clinically approved medications functionally inhibit ASM and are called FIASMAs (functional inhibitors of acid sphingomyelinase). The FIASMA fluvoxamine showed beneficial effects on COVID-19 in a randomized prospective study and a prospective open-label real-world study. Retrospective and observational studies showed favorable effects of FIASMA antidepressants including fluoxetine, and the FIASMA hydroxyzine on the course of COVID-19. The ASM/ceramide system provides a framework for a better understanding of the infection of cells by SARS-CoV-2 and the clinical, antiviral, and anti-inflammatory effects of functional inhibitors of ASM. This framework also supports the development of new drugs or the repurposing of “old” drugs against COVID-19.

## SARS-CoV-2

Severe acute respiratory syndrome coronavirus 2 (SARS-CoV-2) is closely related to the deadly coronaviruses SARS-CoV-1 and Middle East respiratory syndrome coronavirus (MERS-CoV). The 2019 outbreak of coronavirus disease (COVID-19), caused by SARS-CoV-2, has become a public health emergency of international concern [1]. Infection with SARS-CoV-2 often results in mild respiratory tract disease, but a substantial number of patients also experience severe symptoms and pneumonia. A high proportion of critically ill patients require intensive care and ventilator treatment, with a high mortality rate [2]. The total infection death rate is approximately 0.66%, rising sharply to 7.8% in people aged over 80 [3]. This has led to excess mortality in many countries [4]. Risk factors for severe/fatal COVID-19 are advanced age, obesity, chronic respiratory disease, hypertension, cardiovascular disease, kidney disease, cerebrovascular disease, malignancy, and diabetes [5]. Severe COVID-19 courses are characterized by hyperinflammation and cytokine storms, with significantly higher serum levels of interleukin (IL)-6, IL-8, IL-10, IL-2R and tumor necrosis factor (TNF)-alpha [6–8].

The SARS-CoV-2-positive strand RNA genome is packaged within the coated capsid [9]. Cellular infection with SARS-CoV-2 is initiated by the binding of the surface unit S1 of the viral spike glycoprotein to its cellular receptor angiotensin-converting enzyme 2 (ACE2), resulting in the cleavage of the viral spike-protein by the activity of transmembrane serine protease 2 (TMPRSS2) and cathepsin B and L and in viral entry [10–13]. Although the binding of the virus to its receptor has been elucidated in detail [10–12], the changes that occur in the host cell membrane during viral processing and entry are largely unknown. However, membrane changes that mediate viral entry may be a very promising target for preventing infection.

## The acid sphingomyelinase/ceramide system

Surface ceramide is generated by the acid sphingomyelinase (ASM), which is a lysosomal protein that catalyzes the conversion of sphingomyelin into ceramide. Since lysosomes are constantly recycling to the plasma membrane, the ASM can be also found on the cell surface and binds to the outer leaflet of the plasma membrane [14–16]. Surface ASM acts as a signaling molecule and generates ceramide in the outer leaflet of the cell membrane [14–17]. Ceramide molecules are very hydrophobic and spontaneously associate with each other to form small ceramide-enriched membrane domains that fuse and form large highly hydrophobic, tightly packed, and gel-like ceramide-enriched membrane domains termed “platforms” [14, 18, 19]. Thus, the generation of ceramide by the ASM dramatically alters the biophysical properties of the plasma membrane. These large, distinct, ceramide-enriched membrane domains serve to cluster, aggregate and reorganize activated receptor molecules such as CD95, CD40, DR5 or β1-integrin, to name a few [14, 20–24]. Ceramide-rich platforms were also shown to mediate a variety of stress stimuli such as γ-irradiation [17, 25], ultraviolet light [26], or Cu^2+^ intoxication [27], as well as infection of cells with at least some pathogenic bacteria and viruses [15, 28]. The high density of activated receptors upon trapping and clustering in ceramide-enriched membrane domains and the proximity to signaling molecules facilitates and amplifies signaling via the specific receptor, as shown for CD95 [29].

## Functional inhibitors of ASM (FIASMAs)

Since the 1970s, it has been shown that weak organic bases such as desipramine have the potential to inhibit the activity of ASM [30–34]. It has been suggested that ASM is bound to intralysosomal membranes and thus protected from proteolytic inactivation. Weak bases diffuse into lysosomes and are trapped after protonation. This leads to an up to 1000-fold intralysosomal accumulation of weakly basic substances [35]. Weak bases also localize in other acidic subcompartments of the cell membrane and thereby inhibit ASM not only in lysosomes but also in certain domains of the cell membrane. Functional inhibition of ASM requires only a few structural conditions; the molecules need to contain a lipophilic organic ring that integrates into the inner lysosomal membrane, a short spacer and a charged tertiary amine group that displaces ASM from the inner lysosomal membrane, which results in the proteolysis of the enzyme in the lysosomal lumen [36, 37]. Therefore, weak bases do not directly inhibit ASM but lead to functional inhibition of ASM. We have proposed the acronym FIASMA (functional inhibitor of acid sphingomyelinase) for a compound from this large group of drugs [38]. FIASMAs include mono-, bi-, tri- and tetracyclic compounds. All FIASMAs identified so far have at least one basic nitrogen atom, have a medium to high log*P* value, and most of them have a molecular weight below 500. FIASMAs more frequently violate Lipinski’s Rule-of-Five than compounds that do not have effect on ASM, and FIASMAs appear to have good permeability across the blood−brain barrier. Conversely, not all lipophilic weak bases are FIASMAs. This is explained below using the example of chloroquine. We have identified several novel FIASMAs (e.g., fluoxetine, fluvoxamine, maprotiline, nortriptyline, orphenadrine, sertraline, dextromethorphan, emetine and triflupromazine) [33, 34], most of which are U.S. Food and Drug Administration (FDA)-approved known bioactive compounds, most likely minimally toxic and potentially readily available for new clinical applications.

By inhibiting ASM, FIASMAs cause a lower cellular concentration of ceramides. Human studies have shown reduced ceramide concentrations in lung endothelial and nasal epithelial cells with treatment with 25–75 mg/day amitriptyline for 2–4 weeks [39, 40].

The accumulation in lipophilic membranes and acidic intracellular compartments explains the high volume of FIASMA distribution, especially in the lung, the entry organ of SARS-CoV-2. The FIASMA amitriptyline will be described in more detail here as an example. With its high lipophilicity and weak basicity (log*P* = 4.92, pKa = 9.4, www.drugbank.ca), amitriptyline strongly accumulates in tissue compartments, e.g., lysosomes [35], resulting in a high volume of distribution (*Vd* = 16 L/kg, www.drugbank.ca). High tissue concentrations can be detected in all organs. In fact, the highest uptake of amitriptyline among all tissues with a lung/blood concentration gradient of approximately 50 is found in the lungs of mice and rats [41, 42]. In humans, even higher concentration gradients between the lung and blood were found [43, 44]. Due to the particularly high accumulation in the lung, effective antiviral concentrations are likely to be achieved with conventional oral therapy with amitriptyline. Compared with blood, we expect a considerably longer elimination half-life of amitriptyline in lung tissue as a result of the high drug concentration in deep compartments such as lysosomes. We have found this for drugs with comparable physicochemical properties in brain tissue [45].

## The role of the ASM/ceramide system in pathogen infection

The ASM/ceramide system is also significant for pathogen infection. Rhinovirus activates ASM and induces ceramide and the formation of ceramide-enriched membrane domains, which serve as entrances for the virus. FIASMAs block infection by viruses: Both amitriptyline and imipramine block the infection of cells with rhinovirus [28]. A similar mechanism was reproduced for Ebola virus (imipramine, desipramine) [46], measles (amitriptyline) [47] and Japanese encephalitis virus (amitriptyline, imipramine) [48]. Other viruses require the ASM/ceramide system for endosomal escape [49]. The ASM/ceramide mechanism also applies to nonviral infections such as *Pseudomonas aeruginosa* [15], *Staphylococcus aureus* [50] and *Neisseria gonorrhoeae* [51, 52].

## The ASM/ceramide system and SARS-CoV-2

Because some antidepressants are widely used in clinical practice and have a very favorable safety profile, we investigated whether these drugs could be repurposed to treat or prevent infections with SARS-CoV-2. Repurposing is a strategy to develop “old” approved drugs for new clinical indications. The advantages are low development costs, shorter development time and usually higher patient safety [53, 54].

### Preclinical evidence

Infection of epithelial cells with SARS-CoV-2 is initiated by binding of the S protein of the virus to ACE2. Binding is followed by fusion of the viral and cellular membrane, which requires priming of spike by cellular proteases that cleave spike into the S1 and S2 subunits [55]. Spike-protein cleavage is mediated by TMPRSS2, but also by cathepsin B and L [13]. Ceramide may have several functions in the infection with SARS-CoV-2: We have shown that these ceramide-enriched membrane domains trap and cluster ACE2 upon cellular infection with SARS-CoV-2, which is very likely a pre-requisite for signaling via this receptor and therefore a pre-requisite for the infection [56]. It is possible that ceramide-mediated clustering of ACE2 in large membrane domains amplifies signaling via ACE2 and is thereby required for internalization of ACE2 and SARS-CoV-2 into endosomes. However, it might be also possible that ceramide generated within endosomes or on the outer leaflet of the cell membrane upon infection with SARS-CoV-2 binds to cathepsins in endosomes and thereby triggers spike-protein priming and membrane fusion. Previous studies using TNF already demonstrated an activation of cathepsins by ceramide [57]. In line with a direct ceramide−protein interaction, it might be also possible that ceramide binds to and directly activates TMPRSS2 and thereby facilitates membrane fusion. Alternatively, ceramide-enriched membrane domains might trap ACE2 and TMPRSS2 within a small, distinct area of the plasma membrane resulting in a high concentration of TMPRSS2 and thereby S-protein priming, membrane fusion and infection. It is important to note that all of these events are inhibited by amitriptyline or other FIASMAs, since the drugs induce a long-lasting degradation of the protein in lysosomes.

Work from our group [58] finds that SARS-CoV-2 activates the ASM/ceramide system, resulting in the formation of ceramide-enriched membrane domains that serve viral entry and infection by clustering ACE2, the cellular receptor of SARS-CoV-2. Amitriptyline and other FIASMAs inhibit ASM and the formation of ceramide-enriched membrane domains, thereby preventing infection with SARS-CoV-2. The destruction of ceramide-enriched membrane domains by means of anti-ceramide antibodies or neutral ceramidase treatment also prevents infection with SARS-CoV-2. Likewise, genetic downregulation of ASM abrogates infection with SARS-CoV-2. The reconstitution of ceramide in cells treated with a FIASMA, anti-ceramide or ceramidase by the addition of exogenous ceramide restores infection with SARS-CoV-2. In humans, oral application of amitriptyline very efficiently blocks the infection of freshly isolated nasal epithelial cells with SARS-CoV-2 [58]. We obtained comparable results with ambroxol [56]. In summary, our data suggest the use of FIASMA medications such as fluoxetine, amitriptyline, ambroxol, anti-ceramide antibodies and neutral ceramidase for the prevention and treatment of coronavirus disease.

These data were confirmed by an independent study also following the ASM/ceramide approach that demonstrated an inhibition of the infection of cultured epithelial cells with SARS-CoV-2 by the FIASMAs fluoxetine, amiodarone and imipramine in Calu-3 and Vero E6 cells [59]. As mechanisms, the authors describe a lack of acidification in acidic organelles and a change in cholesterol abundance. The observation of the antiviral activity of fluoxetine was also confirmed in other preclinical studies [60, 61]. Taken together, these results show the potentially crucial importance of the ASM/ceramide system as a treatment target in COVID-19, a mechanism likely to be shared by all virus variants [62].

In accordance with these results, activity against SARS-CoV-2 and other coronaviruses was found in a number of cell culture screening studies for different substances that can be assigned to FIASMAs. These studies were either hypothesis-free or pursued hypotheses beyond the ASM/ceramide approach.

In a screening of FDA-approved compounds for anti-SARS-CoV-2 activity, the FIASMAs clemastine and cloperastine were identified [63]. The FIASMAs benztropine, chlorpromazine, clomipramine, emetine, fluphenazine, promethazine and tamoxifen had antiviral effects against SARS-CoV-2 in Vero E6 cells [64]. In a screening of FDA-approved substances against SARS-CoV-2, 24 substances showed antiviral activity in Vero cells, including the FIASMAs cepharanthine and loperamide [65]. In a multistep in vitro screen, the FIASMA promazine was identified as a high-confidence inhibitor of SARS-CoV-2 replication [66]. In addition, recent studies show that fluoxetine inhibited SARS-CoV-2 infections in Vero cells [61] and amiodarone and clofazimine inhibited SARS-CoV-2 infections in iAEC2 cells [67, 68].

A similar antiviral activity of FIASMAs was found against other coronaviruses. An in vitro study on Vero E6 cells showed anti-MERS-CoV and anti-SARS-CoV activity in 27 licensed small-drug molecules, including antipsychotics and estrogen receptor antagonists. Many of these are FIASMAs (astemizole, benztropine, chlorpromazine, clomipramine, emetine, fluphenazine, promethazine, tamoxifen, triflupromazine) [69]. A screening of FDA-approved drugs on Vero cells revealed that the FIASMAs chlorpromazine and loperamide had anti-MERS-CoV activity [70]. In a screen against MERS-CoV replication in Huh-7 cells, the FIASMAs chlorpromazine, promethazine, fluphenazine, astemizole, triflupromazine, clomipramine and tamoxifen demonstrated antiviral activity [71]. The FIASMA emetine exerted in vitro antiviral activity against coronaviruses [72–74]. Clofazimine was active against feline coronavirus in Fcwf-4 cells [75].

### Clinical evidence of the potential usefulness of the FIASMAs fluoxetine, fluvoxamine and hydroxyzine in COVID-19

The notion that FIASMA antidepressants and hydroxyzine inhibit infections with SARS-CoV-2 is also strongly supported by clinical studies.

The first study was a retrospective observational study, indicating a marked beneficial effect of antidepressants on the clinical course of COVID-19. That study was conducted at Greater Paris University Hospitals, France, with 7230 adults hospitalized for laboratory-confirmed COVID-19 between January 24 and April 1, 2020, including 345 patients (4.8%) who received an antidepressant within 48 h of hospital admission at a mean fluoxetine-equivalent dose of 21.6 mg (SD = 14.1) per day [76]. The primary endpoint was a composite of intubation or death and was compared between patients who received antidepressants and those who did not in time-to-event analyses adjusted for patient characteristics (such as age, sex, obesity, and medical comorbidities), clinical and biological markers of disease severity, and other psychotropic medications. The primary analysis was a multivariable Cox model with inverse probability weighting. The results indicated a significant association between antidepressant use and reduced risk of intubation or death (AHR, 0.56; 95% CI, 0.43−0.73, *p* < 0.001). This association was similar in multiple sensitivity analyses. Exploratory analyses also suggested that this association was also significant for selective serotonin reuptake inhibitors (SSRIs) and non-SSRI antidepressants, and for fluoxetine, paroxetine, escitalopram, venlafaxine, and mirtazapine (all *p* < 0.05).

The second study was a prospective randomized placebo-controlled study in which Lenze et al. [77] showed favorable effects of the SSRI and FIASMA fluvoxamine on COVID-19 disease progression in outpatients. Participants randomly received 100–300 mg fluvoxamine (*n* = 80) or placebo (*n* = 72) daily for 15 days. The primary outcome was clinical worsening within 15 days of randomization, defined by meeting the two criteria of (1) a shortness of breath or hospitalization for shortness of breath or pneumonia and (2) oxygen saturation of <92% of room air or a need for supplemental oxygen to achieve an oxygen saturation of 92% or greater. Clinical worsening occurred in 0 of 80 patients in the fluvoxamine group and 6 of 72 patients in the placebo group (absolute difference, 8.7% from the survival analysis; log rank *P* = 0.009). In the fluvoxamine group, there was 1 serious adverse event and 11 other adverse events, while in the placebo group, there were 6 serious adverse events and 12 other adverse events. In summary, in this preliminary study, adult ambulatory patients with symptomatic COVID-19 treated with fluvoxamine had a lower probability of clinical worsening over 15 days compared to those who received placebo.

This observation was confirmed in a third, prospective real-world evidence study, in which the incidence of hospitalization was 0% among 65 persons with COVID-19 who opted to receive fluvoxamine (50 mg twice daily), whereas it was 12.5% among the 48 persons with COVID-19 who declined. At 14 days, residual symptoms persisted in 0% (0 of 65) with fluvoxamine and 60% (29 of 48) with observation [78].

In a fourth study, we investigated the association between the use of the FIASMA hydroxyzine and mortality in patients hospitalized for laboratory-confirmed COVID-19 in a multicenter observational retrospective cohort study involving Greater Paris University Hospitals, France [79]. More than 7000 adults hospitalized for laboratory-confirmed COVID-19 between January 24 and April 1, 2020 were included. Of them, 138 patients (1.9%) had received hydroxyzine during the visit at a mean dose of 49.8 mg (SD = 51.5). The study endpoint was death and was compared between patients who received hydroxyzine and those who did not in time-to-event analyses adjusting for patient characteristics (such as age, sex, and comorbidities), clinical and biological markers of disease severity, and the use of other medications. The results indicated that over a mean follow-up of 20.3 days (SD = 27.5), 994 patients (13.5%) had a primary endpoint event. The primary multivariable analysis with inverse probability weighting showed a significant association between hydroxyzine use and reduced mortality (HR, 0.42; 95% CI, 0.25−0.71; *p* = 0.001), with a significant dose−effect relationship (HR, 0.10; 95% CI, 0.02−0.45; *p* = 0.003). This association was similar in multiple sensitivity analyses. In secondary analyses conducted among subsamples of patients, there was a significant association between hydroxyzine use and a faster decrease in biological inflammatory markers associated with COVID-19-related mortality, including the neutrophil-to-lymphocyte ratio (NLR), the lymphocyte-to-C-reactive protein ratio (LCRP), and circulating IL-6 levels (all *p* < 0.016), with a significant dose−effect relationship for the NLR and LCRP (both *p* < 0.037).

### Clinical evidence of the importance of the ASM/ceramide system as a treatment target in COVID-19

The potential benefit of FIASMA treatments among patients hospitalized for severe laboratory-confirmed COVID-19 was recently explored in an observational multicenter retrospective study [80]. Therein, taking a FIASMA medication upon hospital admission was associated with substantially reduced likelihood of intubation or death. This association was not specific to one FIASMA class (e.g., FIASMA antidepressants) or medication (e.g., fluoxetine). A similar significant association was found in another observational multicenter retrospective study conducted in patients with psychiatric disorders and hospitalized for severe COVID-19 [81]. A retrospective observational study also established a positive association between chronic administration of FIASMA and reduced mortality in COVID-19 hospitalized patients that was significant for the FIASMA amlodipine [82]. Finally, plasma markers of ceramide metabolism were found to be associated with respiratory severity and to correlate with inflammation in 49 patients hospitalized for COVID-19 [83]. The observation of an association between ceramide levels and COVID-19 respiratory distress was also reported in a preprint study involving 52 patients with COVID-19 [84].

Taken together, this clinical evidence suggests that the beneficial effects of certain antidepressants, such as fluoxetine or fluvoxamine, and the H1 antihistamine hydroxyzine, which are FIASMAs [34], in patients with COVID-19 may be mediated by the inhibition of ASM. These findings also support the continuation of FIASMA medications during SARS-CoV-2 infection [80, 81]. While we recognize that retrospective observational studies are subject to bias, they are nevertheless examples of how molecular insights can quickly generate testable clinical hypotheses and help prioritize candidates for prospective clinical trials or future drug development.

The potential negative effects of chloroquine and hydroxychloroquine in COVID-19 are also compatible with the significance of the ASM/ceramide system described above. Earlier approval for chloroquine and hydroxychloroquine for the treatment of COVID-19 has been revoked by the FDA [85] because of a potential increased mortality under hydroxychloroquine and no benefit of chloroquine in inpatients [86]. Chloroquine and hydroxychloroquine are cationic amphiphilic substances with strong accumulation in acidic intracellular compartments. The antimalarial effect of chloroquine is apparently at least partially based on its alkalinizing effect on acidic intracellular compartments [87]. However, chloroquine does not result in functional inhibition of ASM [32, 34, 88]. Instead, chloroquine results in increased ceramide content in lung cells of mice [89], possibly by the inhibition of acid ceramidase [90], which is also located in acidic intracellular compartments. The increased ceramide abundance in lung cells could explain its potentially negative effect in the treatment of COVID-19.

## The ASM/ceramide model of SARS-CoV-2 infection and COVID-19

Considering SARS-CoV-2 infection and COVID-19 from the perspective of the ASM/ceramide system provides a framework for a deeper understanding and for the development of testable hypotheses. Here are a few examples:

1. Substances that increase cellular ceramide abundance should have an unfavorable effect on the course of the disease. This was explained above using the example of hydroxychloroquine, but these findings should be examined in more detail.
2. Conversely, interventions that lower ceramide levels should have a beneficial effect on COVID-19 disease progression. In a randomized controlled trial, rosuvastatin dose-dependently lowered plasma ceramide levels, independent of cholesterol levels [91]. This may explain the favorable disease outcome of COVID-19 patients taking rosuvastatin [92].
3. In cell culture, subthreshold concentrations of FIASMAs have an additive inhibitory effect on ASM [34]. Therefore, if a low dose of a single FIASMA does not affect the course of COVID-19, the combination of two or more FIASMAs, each at a low dose, may be effective.
4. Clinical data can now be analyzed for the effects of FIASMAs vs. non-FIASMAs on the course of COVID-19. The course of patients under antidepressant therapy should be more favorable with FIASMA antidepressants than with non-FIASMA antidepressants. The patients in both groups would be comparable because they all received antidepressants. Similarly, the clinical course of COVID-19 in patients treated with antipsychotics or cardiotropic medications could be investigated by comparing FIASMA and non-FIASMA-antipsychotics or cardiotropic medications [80, 81].
5. Agonistic modulation of the sigma1 receptor pathway has a beneficial effect in preclinical inflammation and sepsis models [93] and has attracted attention in analyses of SARS-CoV-2−host interactions [63, 94]. Fluvoxamine is a sigma1 receptor agonist [95, 96]. Although it is possible that the favorable effects of fluvoxamine in the prospective study in outpatient COVID-19 patients [77] are due to this effect, fluvoxamine also acts as a FIASMA [34]; this mechanism may also explain its beneficial effect on the course of COVID-19 disease. Based on preclinical evidence for an effect of sigma1 receptor agonists on inflammation and cytokine secretion, compounds with an intrinsically dual mechanism of action, i.e., acting both as a sigma1 agonist and a FIASMA (e.g., fluvoxamine or fluoxetine) should act better on COVID-19 than FIASMAs with no or antagonistic effect on the sigma1 receptor (e.g., sertraline) [97]. Conversely, it should also be investigated whether sigma1 agonists without simultaneous effects on the ASM/ceramide system can also be successfully used in COVID-19.
6. Remdesivir alone shows limited efficacy on COVID-19 and is no longer recommended for therapeutic use according to the WHO [98]. However, according to in vitro data, it is quite conceivable that the combination of remdesivir with a FIASMA will provide additional benefits. This has been shown for the combinations remdesivir−emetine [72] and remdesivir−fluoxetine [99].
7. Plasma ceramides are elevated in sepsis patients and predict sepsis-associated mortality [100]. Acute systemic inflammation highly upregulates secretory sphingomyelinase [101–103]. Preclinical studies show favorable effects of genetic downregulation or the application of the FIASMAs amitriptyline or desipramine in sepsis models [104, 105]. Therefore, the role of ceramide in the initial development and further progression of COVID-19-associated sepsis should be investigated. Plasma markers of ceramide metabolism were found to be associated with respiratory severity and to correlate with inflammation in 49 patients hospitalized for COVID-19 [83, 84]. It is tempting to speculate that FIASMAs also have a beneficial effect on the course of sepsis in COVID-19.
8. Currently, hyperinflammation in COVID-19 is treated with broad-spectrum corticosteroids or specific monoclonal antibodies. While corticosteroids have a substantial effect among critically ill patients with COVID-19 [106, 107], the effect of specific therapies, for example, tocilizumab as a monoclonal humanized antibody against IL-6, appears to be significantly smaller [108]. ASM is a critical regulator of IL-6 production [109] and plays an important role in TNF-alpha signaling [110]. Interestingly, antidepressants reduce peripheral cytokine levels of IL-6, TNF-alpha, IL-10 and CCL-2 in the usual treatment of patients with major depression [111]. Future clinical studies must show whether this cytokine-reducing effect is due to FIASMAs among the antidepressants. Furthermore, future studies must show whether FIASMAs also have a beneficial effect on cytokine release in patients with COVID-19. At least in a retrospective evaluation, the FIASMA hydroxyzine was associated with a faster decrease in biological inflammatory markers associated with COVID-19-related mortality, including the neutrophil-to-lymphocyte ratio, the lymphocyte-to-C-reactive protein ratio, and circulating IL-6 [79]. FIASMAs could thus not only inhibit the entry of SARS-CoV-2 but also alleviate the cytokine storm in severe COVID-19 courses. Thus, not only prophylactic and early antiviral use of FIASMAs is possible but also a therapeutic approach for later severe courses of COVID-19.
9. Risk factors for an unfavorable course of COVID-19 can be explained in part by the ASM/ceramide system. Elevated ceramide levels have been associated with higher age [112–116], hypertension [117] and obesity [118], three of the major risk factors for the development of severe infections with SARS-CoV-2 [5]. It is therefore tempting to speculate that the elevated ceramide levels associated with these risk factors sensitize cells to infection with SARS-CoV-2, thereby contributing to the development of severe infections.
10. Complications in the course of COVID-19 may also be partially explained by activation of the ASM/ceramide system. COVID-19 infection with SARS-CoV-2 infection is associated with a hypercoagulable state. Activation of ASM induces blood coagulation via decrypting of tissue factor TF [119–121]. FIASMAs may therefore prevent the thromboembolic complications of SARS-CoV-2 infection.

## Conclusion

We have described the role of the ASM/ceramide system in the infection of cells with SARS-CoV-2. Pharmacological or genetic downregulation of ASM protects against infection. The neutralization or degradation of ceramides on the cell surface also protects against infection. The addition of exogenous ceramide after the downregulation of ASM by pharmacological inhibition restores infection with SARS-CoV-2. Thus, ceramide on the cell surface is necessary for infection with SARS-CoV-2. The ASM/ceramide system may be important not only for virus entry into the cell but also for the release of cytokines such as IL-6. Antidepressants reduce the level of proinflammatory cytokines in preclinical studies and patients with major depressive disorder. Importantly, reduced IL-6 levels were also found in COVID-19 patients treated with the FIASMA hydroxyzine. The ASM/ceramide system can help to explain the typical risk factors for a lethal COVID-19 course since ceramide abundance is increased in advanced age, hypertension or obesity, and the increased rate of thromboembolic complications in the setting of COVID-19 can also be explained by the ASM/ceramide system. The ASM/ceramide system may also help to explain the negative effect of chloroquine in the therapy of patients with COVID-19.

The ASM/ceramide system can be downregulated with FIASMAs. Many FIASMAs have been approved by the FDA for use in humans, and many have been used for decades as well-tolerated and safe drugs. Our preclinical data on the beneficial effects of FIASMAs are supported by a prospective clinical study with fluvoxamine and retrospective observational studies of patients receiving or not receiving antidepressants or hydroxyzine. The data from Lenze et al. [77] show favorable effects during the early stages of COVID-19, and the data from Hoertel et al. [79, 80] show favorable effects during the later stages of COVID-19. FIASMAs may thus be effective over the entire course of the disease, from infection of the cells through hyperinflammation to sepsis. This is probably due to different mechanisms, such as virus entry into the cell, cytokine secretion and sepsis, which are, however, jointly regulated by the ASM/ceramide system.

In this review, we have also highlighted how the ASM/ceramide approach allows the formulation of testable hypotheses for the development, repurposing and application of anti-COVID-19 drugs.

In summary, the COVID-19-ASM/ceramide system helps us to understand (1) the entry of SARS-CoV-2 into cells; (2) hyperinflammation and increased levels of proinflammatory cytokines, such as IL-6; (3) mortality in severe sepsis; (4) risk factors for severe disease progression, such as age, hypertension and obesity; (5) thromboembolic complications; and (6) the beneficial effects of FIASMAs during both early and later stages of COVID-19. The COVID-19-ASM/ceramide system also supports the development of drugs against COVID-19, for example, by repurposing the FDA-approved FIASMAs fluoxetine or fluvoxamine, which display high in vitro inhibition effect on ASM, showed potential positive effects at usual antidepressant doses, and are easy to use, including high safety margins, good tolerability, widespread availability and low cost [80, 122].

## Disclaimer

The information contained in this review is provided for reference only and should not be used as a substitute or replacement for diagnosis or treatment recommendations or other clinical decisions or judgment
